# Functional Gastrointestinal Disorders and Childhood Obesity: The Role of Diet and Its Impact on Microbiota

**DOI:** 10.3390/nu17010123

**Published:** 2024-12-30

**Authors:** Valeria Calcaterra, Hellas Cena, Federica Loperfido, Debora Porri, Sara Basilico, Cassandra Gazzola, Cecilia Ricciardi Rizzo, Maria Vittoria Conti, Giovanni Luppino, Malgorzata Gabriela Wasniewska, Gianvincenzo Zuccotti

**Affiliations:** 1 Department of Internal Medicine and Therapeutics, University of Pavia, 27100 Pavia, Italy; 2 Pediatric Department, Buzzi Children’s Hospital, 20154 Milano, Italy; cassandra.gazzola@unimi.it (C.G.); gianvincenzo.zuccotti@unimi.it (G.Z.); 3 Laboratory of Dietetics and Clinical Nutrition, Department of Public Health, Experimental and Forensic Medicine, University of Pavia, 27100 Pavia, Italy; hellas.cena@unipv.it (H.C.); federica.loperfido@unipv.it (F.L.); sara.basilico01@universitadipavia.it (S.B.); cecilia.ricciardirizzo@unipv.it (C.R.R.); mariavittoria.conti@unipv.it (M.V.C.); 4 Clinical Nutrition and Dietetics Unit, ICS Maugeri IRCCS, 27100 Pavia, Italy; 5 Pediatric Unit, AOU Policlinico “G. Martino”, 98122 Messina, Italy; debora.porri@gmail.com (D.P.); giovilup97@gmail.com (G.L.); mwasniewska@unime.it (M.G.W.); 6 Department of Human Pathology of Adulthood and Childhood, University of Messina, 98122 Messina, Italy; 7 Department of Biomedical and Clinical Science, University of Milano, 20157 Milano, Italy

**Keywords:** functional gastrointestinal disorder, functional bowel disorder, children, adolescent, obesity, childhood obesity, diet, gut microbiota, biotic

## Abstract

Introduction Emerging evidence suggests an association between obesity and Functional Gastrointestinal Disorders (FGIDs). Childhood obesity and FGIDs share many common features, such as high prevalence in the pediatric population, risk factors related to diet and lifestyle, gut microbiota impairments, and psychological distress. This narrative review aims to summarize the main evidence regarding FGIDs in childhood obesity, with a specific focus on the role of diet and its impact on the microbiota. Additionally, the review highlights potential common-ground solutions for preventing and managing both obesity and FGIDs. Methods A comprehensive PubMed search was conducted. Keywords used included terms related to children and adolescents, obesity, functional gastrointestinal disorders, and microbiota. Results The review emphasizes the importance of holistic, multidisciplinary approaches to managing symptoms. In addition to nutrition education, physical activity, and medical care, complementary strategies such as psychological interventions and personalized dietary modifications (e.g., low-FODMAP and fiber-enriched diets) are critical. Given the interplay between gut microbiota alterations, obesity, and FGIDs, microbiota modulation through probiotics, prebiotics, and integrative support shows significant promise. However, the variability in current evidence underlines the need for robust longitudinal studies to develop standardized protocols and maximize treatment efficacy. Conclusions Bridging gaps in knowledge and practice with an integrated, evidence-based framework could improve patient outcomes and deepen understanding of the complex relationship between metabolic and gastrointestinal health in children and adolescents.

## 1. Introduction

Functional Gastrointestinal Disorders (FGIDs) encompass a range of chronic gastrointestinal symptoms, often accompanied by pain, with no identifiable medical condition. Functional bowel disorders are a type of functional gastrointestinal disorders including conditions characterized by symptoms originating from the middle or lower gastrointestinal tract [1]. Main Functional Bowel Disorders (FBDs) in children and adolescents are Irritable Bowel Syndrome (IBS) and Functional Dyspepsia (FD), (included among Functional Abdominal Pain Disorders by Rome IV criteria) and Functional Constipation (FC) (included among Functional Defecation Disorders by Rome IV criteria) [2].

Nowadays, well-defined epidemiological data about the prevalence of FGIDs in pediatric patients is still lacking. According to a systematic review, prevalence rates of FGIDs in children and adolescents could range from 9% to 29% [3]. Notably, IBS is one of the most widespread conditions among FGIDs. A meta-analysis reported that the pooled global prevalence of IBS reached 8.8% in children and adolescent aged 4–18 years [4]. Rather, the estimated prevalence of IBS in the European Mediterranean region is 4% in the same population [5]. Regarding FC, a study reported a pooled global prevalence in children equal to 14.4%, specifically in Europe at 8.3% [6]. Instead, the prevalence of FD in children is estimated to range from 3% to 27%, and it poses a significant economic burden on healthcare systems. Children with FD who show partial or no response to treatment often incur higher medical costs, further contributing to healthcare expenditures [7]. FGIDs in children imply relevant healthcare costs and loss of economic productivity for parents or caregivers [8,9]. For example, globally, FC accounts for 25% of pediatric gastroenterologist consultations and 3% of all general pediatric visits [6].

IBS diagnosis is symptoms-based. The main symptom is abdominal pain, which can be related to defecation or/and diarrhea, constipation, or both conditions [2]. It has been hypothesized that IBS may arise from disruption of the brain-gut axis. There is an ongoing debate about whether the primary dysfunction onsets in the gut (e.g., accumulation of inflammatory cells in the intestinal mucosa) or in the central nervous system (e.g., increased release of corticotrophin releasing factor, CRF) [10]. The disruption of the brain-gut axis leads to different pathophysiological changes, such as visceral hypersensitivity, gastrointestinal dysmotility, increased gut permeability, and intestinal low-grade inflammation with the involvement of microbiota [10,11]. A child’s psychological distress can be associated with IBS [2,11]. Indeed, risk factors for IBS include excess worry, anxiety, and depression, but also gastrointestinal infections and diet (e.g., important assumption of spicy or fried food) [10].

Patients affected by FD complain one or more of the following bothersome symptoms, such as postprandial fullness, early satiation, and epigastric pain or burning not in concomitance with defecation, at least four days per month [2]. Different pathophysiological pathways are hypothesized as responsible for FD development such as motor abnormalities (e.g., delayed gastric emptying or antral hypomotility), visceral hypersensitivity to gastric distension, genetic predisposition, low-grade inflammation and psychosocial factors (e.g., anxiety) [2,12].

FC is a disorder characterized by infrequent defecations associated with painful and hard bowel movements, retentive behavior, presence of a large fecal mass in the rectum or large diameter stools able to clog the toilet, or episodes of overflow fecal incontinence [2]. Pathophysiological mechanisms are multifactorial and include stool withholding, dietary habits, physical activity, and anorectal dysfunctions [6]. A child’s avoiding defecation instinct because of pain or social reasons is the most important trigger of the disorder [2].

According to WHO, in 2022, 160 million children and adolescents aged 5–19 years were living with obesity worldwide [13]. Currently, childhood obesity is a global health issue, exhibiting characteristics of a pandemic. Furthermore, causes of childhood obesity can include individual determinants, as well as social and environmental factors. Indeed, the “obesogenic environment” plays an important role in the development and perpetuation of obesity among children and adolescents. Specifically, two of the main drivers of childhood obesity are a dietary pattern characterized by energy dense nutrient poor foods and a sedentary lifestyle [14]. Obesity in children and adolescents aged 4–18 years is a significant public health issue, as it increases the risk of cardiometabolic and psychosocial comorbidities during childhood and adolescence, and is associated with premature mortality in adulthood [14].

Moreover, emerging evidence suggests an association between obesity and FGIDs [15,16]. Childhood obesity and FGIDs share many similar features such as high incidence in the children population, risk factors related to diet and lifestyle, gut microbiota impairment, and psychological distress. Studies addressing this issue are heterogeneous, especially in terms of FGIDs diagnostic criteria considered and cohorts analyzed [17].

Our review aims to summarize the main evidence regarding FGIDs in childhood obesity, with a specific focus on the role of diet and its impact on microbiota. Additionally, this review could contribute to pointing out common-grounded solutions for preventing and treating both obesity and FGIDs.

## 2. Methods

This is a narrative review to explore the association between childhood obesity and FBDs. To accomplish this, a comprehensive search of the PubMed database was conducted, focusing on English-language articles published over the past 25 years.

The review included a variety of publication types, such as original research studies, systematic and narrative reviews, meta-analysesand longitudinal studies, while excluding case reports, case series, letters, and commentaries. Relevant studies exploring specific interventions, including probiotics, prebiotics, and dietary modifications, were included if they contributed to understanding pediatric FBDs and obesity.

The search strategy employed specific keywords, both individually and in combination, including: child OR children OR childhood OR adolescent AND obesity OR obese OR overweight AND functional gastrointestinal disorder* OR functional gastrointestinal disease* OR constipation OR functional constipation OR dyspepsia OR abdominal pain OR functional bowel disorder* OR functional bowel disease* AND biotic OR probiotic* OR prebiotic* OR synbiotic OR fiber AND microbiota OR gut microbiota.

Articles were initially screened based on titles (*n* = 3275) and abstracts (*n* = 2432), and those deemed relevant were subjected to a full-text review for further evaluation (*n* = 130). Selected studies (*n* = 120) were critically assessed to ensure a comprehensive understanding of the topic. The draft of this review was collaboratively prepared, thoroughly reviewed, and approved by all co-authors to ensure the accuracy and depth of the final analysis.

**Figure 1:**
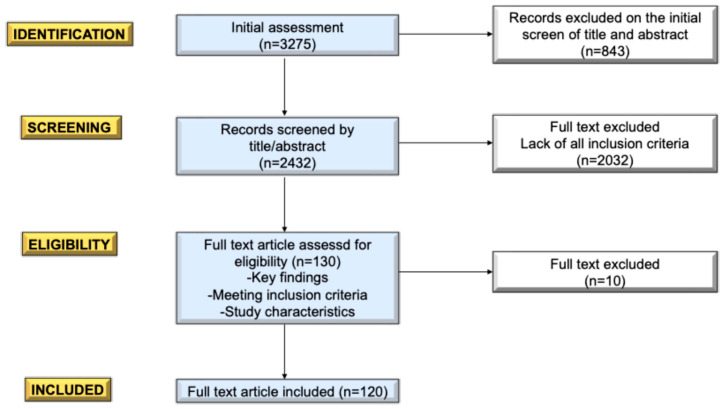
Flowgram of the selection of the manuscripts.

The process for manuscript selection is summarized in Figure 1.

## 3. Functional Bowel Disorders in Children with Obesity: State of the Problem

In the last twenty years, childhood obesity has reached epidemic proportions. In 2006, 16.6% of children and adolescents aged 2 to 19 had a weight at or above the 95th percentile for age. More than 7% of children and adolescents had obesity in 2016 compared with less than 1% in 1975 [18].

According to global health estimates provided by World Health Organization (WHO) 38.2 million of children under the age of 5 years present overweight or obesity in 2019 [13]. The rapidly increasing prevalence of obesity in children is the most important problem facing pediatricians today [19,20,21].

In addition to the well-known association with cardiovascular disease and diabetes, recent studies have reported an association between obesity and FGIDs [15,16]. Teitelbaum et al. [22] found a significantly higher prevalence of obesity in children with FGIDs compared to the control group; in a retrospective study evaluating the association with obesity in children and adolescents referred to a pediatric gastroenterologist, 23% of patients affected by FC were living with obesity, while 24.8% of patients with IBS were affected by obesity. The study also took into consideration recent evaluations regarding gastrointestinal motility disorders, delayed gastric emptying and alteration of the gastric antrum, or functional dyspepsia, were also found in children with Functional Abdominal Pain (FAP). The association between these disorders and overweight has been established through prior research [23]. Greater gastric distension in overweight children can lead to a reduction in the tone of the stomach muscle wall, altering the sensitivity of the mechanoreceptors and also causing an abnormal perception of satiety [24]. This retrospective study also highlighted a higher prevalence of overweight/obesity in females, who are more subdued to gastrointestinal motility disorders than males. It could be caused by the action of ovarian hormones or by gender differences in the microbiome, but it’s still being studied [25].

Galai et al. [26] in a retrospective study reported that adolescents with FAP had higher prevalence of overweight/obesity compared to controls (39.5% vs. 30%).

Tambucci et al. found a significantly higher prevalence of FGIDs in children and adolescents living with obesity or overweight rather than in normal-weight children [27]. The study enrolled 103 children and 115 controls, all patients were evaluated by an endocrinologist and a gastroenterologist and Rome III questionnaires were used for the diagnosis of functional gastric disorder. A statistically significant difference was noted between the two groups for functional constipation, functional dyspepsia, and irritable bowel syndrome, but no statistically significant difference was observed for FAP. Notably, the prevalence of FC and IBS was respectively 18.44% and 10.67% in children with overweight/obesity.

The study by Phatak et al. [28] found a higher prevalence of FBDs in children with obesity/overweight (16.1%) compared to normal weight subjects (6.9%). The study was performed on children between ages of 4 and 18 years from the Yale Pediatric Primary Care clinic, FBDs studied were FC, FAP and IBS. A total of 450 children were recruited and almost half of the children with obesity/overweight had at least one functional gastrointestinal disorder.

Regarding FC, an association between obesity and increased prevalence of constipation has been noted [29]. A retrospective study was performed in June 2004 on 719 children, between the ages of 4 and 18 years, with chronic functional constipation; the control group consisted of 930 children in a pediatric clinic. Prevalence of obesity was significantly higher in constipated children (22.4%) compared with control children (11.7%), especially in males. The higher prevalence of obesity may be a result of dietary factors, activity or hormonal influences and needs additional evaluation.

A review published in 2022 by Zia JK et al. [30] highlights how it is necessary to implement studies on the pediatric population to determine the risk factors of IBS and other functional disorders, however finding a statistically significant association between obesity and functional gastrointestinal disorders.

Therefore, there is certainly a link between overweight and functional gastrointestinal disorders, although the results on each of the individual disorders are still conflicting according to different studies. The heterogeneity in findings across FBD subtypes, such as the stronger association between obesity and constipation compared to IBS, may stem from differences in gut motility, dietary patterns, or hormonal influences. Future research should stratify pediatric populations by FBD subtype to clarify these dynamics [29,31].

As reported, not only can obesity increase the prevalence of FBDs, but it can also modify their outcome. In this regard we mention a prospective cohort study from 2011 [32], which included 188 children, of which 20% present obesity. The results of the study demonstrated a statistically significant difference between normal weight patients and patients with obesity regarding the frequency of abdominal pain, intensity and limitation to daily activities.

Although obesity is known to increase the risk of reflux esophagitis, pediatric data on the association between obesity and gastroesophageal reflux are less clear: Elitsur et al. [31] showed that, within a group of children with reflux, esophagitis was observed in 65% of normal weight cases, in 69% and 68% of children with overweight with obesity respectively, with no significant difference between these groups. Similarly, Patel et al. [33] did not find a significant difference in the prevalence of reflux esophagitis among normal weight patients (23.9%) and overweight (24.5%). On the contrary, in adults the difference between the normal weight and overweight groups was statistically significant.

The pathophysiology of FBDs remains uncertain, with multifactorial underlying mechanisms. Evidence suggests a relationship between the microbiome, obesity, and FBDs, which could be leveraged to improve these gastrointestinal disorders. Notably, improvements in eating habits appear to be closely linked with enhancements in the microbiome, leading to better BMI outcomes and relief from functional disorders [34,35]. Current research indicates that factors such as diet, exercise, stress, and medications significantly influence gut microbiota alterations. Exploring this relationship presents a promising therapeutic approach [36,37].

A diet high in fat is risky for many reasons, some studies have highlighted that it is even responsible for greater malignant tumor progression, through the destruction of the intestinal microbiota. Not only does overweight alter the intestinal microbiota, but at the same time the inflammatory alteration induced by a pathological microbiota alters hypothalamic gene expression, vagus nerve activity and angiogenesis, creating a vicious circle. Some recent studies have compared the intestinal microbiome of normal weight subjects with patients with obesity and have noted that biodiversity is greatly reduced in subjects, also reducing the ability to resist external infections and increasing inflammation and intestinal permeability [38,39,40].

The proper functioning of the intestinal barrier is essential to prevent bacterial infiltration. Many pathologies, including IBS and various functional intestinal disorders, are characterized by an altered, more permeable intestinal barrier, which increases bacterial translocation. Individuals with IBD have significantly higher intestinal permeability compared with healthy controls; that suggests that one of the mechanisms underlying disease pathogenesis in IBD is damage to the structural barrier of the GI tract. Individuals with IBD have elevated levels of circulating proinflammatory mediators and this systemic inflammation has been suggested to be due to MT because elevated serum levels of LPS, bacterial DNA and LBP can be detected [41,42,43,44,45,46,47,48].

As detailed in the following sections, in the pediatric field, combined strategies, such as diet, nutritional education, use of biotics and integrative support, serve as cornerstone approaches for achieving improved symptom management.

In Table 1 the main papers referred to state of the problem about FGIDs in children and adolescents with obesity are reported.

## 4. Diet, Nutritional Education and Support

### 4.1. The Role of Diet in FBDs

Among pathogenic mechanisms involved in FBDs, aspects related to nutrition—such as diet type, eating behaviors, gastrointestinal tract function, motility, and intestinal inflammation—play a significant role [49,50]. Currently, the pathophysiology of FBDs remains uncertain, with underlying mechanisms that are multifactorial. Among these mechanisms, aspects related to nutrition—such as diet type, eating behaviors, gastrointestinal tract function, motility, and intestinal inflammation—play a significant role [49,50]. In addition to the conventional treatment options, specific dietary patterns have shown potential benefits. These include the low-FODMAP (fermentable oligosaccharides, disaccharides, monosaccharides, and polyols) diet, as well as diets that restrict fructose, lactose, or gluten [4,51,52,53,54]. A recent systematic review and meta-analysis by Hua et al. [55] aimed to compare the effectiveness of various dietary approaches in children and adolescents with functional abdominal pain disorders but found no significant differences between the dietary treatments. In the following paragraphs, we summarize the characteristics and supporting evidence for each dietary approach.

#### 4.1.1. FODMAP Diet

Fermentable oligosaccharides, disaccharides, monosaccharides, and polyols (FODMAPs) are short-chain carbohydrates that are poorly absorbed in the small intestine, which can lead to bloating and gas. FODMAPs are found in various food groups, particularly in vegetables such as beetroot, broccoli, Brussels sprouts, cabbage, fennel, garlic, leek, onion, pumpkin, shallot, courgette, and squash, as well as in cereals like corn, rye, and wheat when consumed in large quantities [56].

Among legumes, those with the highest FODMAP content include beans, chickpeas, lentils, peas, red beans, and soybeans. FODMAPs are also present in certain fruits, such as apricots, bananas, blueberries, cranberries, currants, quinces, grapefruits, melons, persimmons, plums, pomegranates, and watermelons, as well as in dried fruits like almonds, cashews, hazelnuts, and pistachios [57].

A low-FODMAP diet is widely used to manage irritable bowel syndrome (IBS) in adults [58]. However, given the broad range of foods to limit, this diet may be challenging for pediatric patients. In 2022, the ESPGHAN (European Society for Pediatric Gastroenterology Hepatology and Nutrition) published a Position Paper on the use of a low-FODMAP diet in children [59], concluding that further evidence is needed to support its use in pediatrics, with some promising findings for IBS. Emerging evidence suggests that dietary interventions, such as low-FODMAP diets, modulate microbiota diversity and fermentation patterns, which can alleviate symptoms like bloating and abdominal pain. However, the long-term impact on microbiota composition and pediatric health remains underexplored [59].

A recent systematic review [60] examined the effects of a low-FODMAP diet on FBDs in children, but the results were insufficient to support specific therapeutic recommendations, except for irritation bowel syndrome, aligning with ESPGHAN’s guidelines [59].

This year, a trial evaluated the impact of a low-FODMAP diet on health-related quality of life in a sample of Egyptian children [61]. Researchers enrolled 84 children aged 5–15 years, randomly assigned to either a low-FODMAP or a standard diet group. Each participant received a list of foods to include and avoid (high-FODMAP foods) and followed dietary advice for six weeks [61]. Weekly assessments included a visual analogue scale (VAS) for pain severity, the Pediatric Quality of Life Inventory (PedsQL), and the GI Symptoms Module Scale.

Results indicated a significant reduction in abdominal pain severity, improved gastrointestinal symptoms, and an enhanced health-related quality of life in children who adhered to the low-FODMAP diet [61]. Interestingly, none of the studies analyzed in Katzagoni et al.’s systematic review reported high health-related quality of life scores [60].

Another recent study, conducted by Tenenbaum et al. [62] in 2024, explored whether quality of life could predict adherence to the low-FODMAP diet in children with FBDs. They found a positive association between quality of life and adherence to the diet. Given the complexity of this dietary strategy, close nutritional follow-up is essential not only to prevent nutrient deficiencies but also to reduce stress in the child by providing recipes or alternatives to maintain dietary variety, thereby improving adherence. However, further studies are needed to clarify the effectiveness of the low-FODMAP diet in children with FBDs.

#### 4.1.2. Fructose or Lactose-Restricted Diet

Fructose and lactose malabsorption are also potential contributors to symptoms associated with FBDs, as both can lead to bacterial fermentation in the intestinal lumen, although through different mechanisms [63,64]. In clinical practice, lactose- and fructose-free diets have been proposed to help manage FBDs symptoms. The aforementioned systematic review [60] gathered evidence regarding this approach, but unfortunately, there is currently insufficient data to recommend lactose- or fructose-free diets universally. While some patients may experience symptom relief with dietary restrictions, further studies are needed to clarify factors such as individual tolerance levels and the threshold amounts that can be consumed without discomfort.

Regarding lactose, it is important to note that the ESPGHAN committee published a position paper on the use of infant formulas in treating functional gastrointestinal disorders [65]. They concluded that there is limited evidence supporting the use of specialized formulas and emphasized that breastfeeding should not be discontinued in favor of formula feeding.

Another noteworthy consideration is that lactose-free diets in children might lead to reduced protein and calcium intake, potentially resulting in shorter stature and lower bone mineral density [66]. However, this claim remains controversial [67,68,69], and further large-scale studies are needed to clarify the relationship between growth outcomes and a lactose-free diet.

In contrast, the link between excess fructose intake and childhood obesity, along with associated conditions such as Nonalcoholic Fatty Liver Disease (NAFLD), is well-established [70,71]. Fructose is present not only in fruits and certain vegetables (e.g., tomatoes) but also as a common sweetener in fruit juices, sodas, and processed sweets. Even though evidence does not currently support fructose restriction as a treatment for FBDs symptoms, it is advisable for children to significantly reduce their intake of these fructose-rich products [72].

Lastly, for managing chronic constipation, the use of certain sugars such as cane sugar, fig syrup, and blackstrap molasses has also been explored as alternative remedies [73,74,75]. Beleli et al. [73] showed that 4′-galactooligosaccharides derived from sugars can improve constipation symptoms in pediatric populations, highlighting their potential as a dietary intervention. Similarly, Dehghani et al. [74] conducted a randomized controlled trial comparing blackstrap molasses with polyethylene glycol, and despite no significant differences between groups, both treatments were shown to have beneficial effects and may represent well-tolerated options for pediatric functional constipation. Additionally, Tajik et al. (2018) explored the use of red sugar versus fig syrup, concluding that both showed promising results as natural treatments for children with functional constipation [75]. However, the heterogeneity of the available evidence, including differences in study design, population characteristics, dosages, and outcome measures, prevents us from making definitive recommendations. However, the heterogeneity of available evidence prevents us from making definitive recommendations.

#### 4.1.3. Gluten-Free Diet

The final dietary approach for managing FBDs is a gluten-free diet. Gluten intake has often been linked to the development of FBDs and worsening of symptoms, although this connection remains unclear [76]. Currently, evidence on the effects of gluten exclusion for FBDs symptoms in children is limited. According to the recent systematic review mentioned earlier [60], only three trials have investigated this relationship [77,78,79], each with varying study designs, interventions, and outcomes. As a result, there is currently no support for the use of a gluten-free diet in managing FBDs in children.

A gluten-free diet requires the complete elimination of gluten-containing grains, such as wheat, barley, rye, and their derivatives. Additionally, gluten can be added as an ingredient in processed foods. Cross-contact during both industrial and home preparation can also lead to contamination, potentially introducing gluten traces in foods that would otherwise be gluten-free.

This potential for contamination might contribute to a finding from a study by Fioti et al. [80], where over 50% of adolescents surveyed expressed concern that a gluten-free diet restricts their food choices. Alongside psychosocial effects, economic implications are also noteworthy: gluten-free foods are often more expensive and less accessible than gluten-containing alternatives [81]. Further studies are necessary to clarify whether a gluten-free diet could be beneficial for children with FBDs.

### 4.2. Nutritional Education and Additional Strategies for Managing FBDs in Children with Obesity

The management of FBDs and obesity involves a unique approach because of the strong pathological correlations of these disorders. Nutritional education and lifestyle management are the primary therapeutic approaches to consider, alongside providing reassurance to the patient and their family. Patients should receive a clear understanding of the diagnosis, a full explanation of the link between overweight and FBDs and be told what organic diseases have been excluded. Exclusion of organic diseases, setting realistic therapeutic goals and sharing the therapeutic decision with the patients give them more satisfaction, better adherence to the outlined therapy and reduce the drop-out [60].

Nutritional education provides elementary rules that should be practiced daily in addition to any indication to specific dietary regimens [55]. The different dietary interventions are characterized by the absence of significant differences in terms of efficacy and safety [55]. The process of deciding which therapy to use for everyone should be guided by a thorough evaluation conducted by the dietitian, with patients actively participating in this process [82]. Moreover, food precautions should be applied in the management and prevention of FBDs [55]. Fiber supplementation in daily practice could be considered because of fibers leads to higher treatment success with minimal side effects [83]. Fiber improves regularity by stimulating bowel movements and can help regulate gastrointestinal symptoms. They also act as prebiotics, supporting gut health and digestion. Additionally, fiber retains water in the stool, promoting texture while promoting a feeling of fullness that can help with weight management [84]. Despite the positive effects, evidence does not indicate the use of fibers supplements and extra fluid intake in the treatment of specific FBDs such as functional constipation [85,86].

Dietary management is the crucial key in controlling functional gastrointestinal disorders symptoms. In several studies, dietary education was provided by a dietitian with verbal instructions or written material [87]. However, given the significant differences in nutrition education and the lack of specialized instructional materials, further efforts are needed to establish a consistent and effective method of educating patients about dietary interventions. Adherence to diet could allow resolution and control of FBDs [88]. Several aspects may represent barriers to adherence: the length of dietary intervention, individual preferences, attitudes towards certain food and diets, nutritional knowledge, physical and social factors [89,90]. Family dynamics and parenting style can influence health care (including approach to decision-making, communication with clinician, medication adherence) and adherence outcomes in pediatric functional constipation. To improve medical regimens and achieve better outcomes, it is essential to study, understand, and incorporate family dynamics into the treatment approach for pediatric constipation [91]. To maximize effectiveness of nutritional interventions and adherence, it is relevant to find ways to assess and address these burdens during clinical contests and nutritional education [89,90,92].

Patient education about physical activity is necessary for the prevention and treatment of FBDs. The role of physical activity in the management of functional constipation is varied. Few studies are conducted on pediatric patients [93,94,95,96], but many studies conducted in adulthood underline the importance of physical activity in the treatment of FBDs [97,98,99].

A negative correlation between physical activity and functional constipation has been demonstrated in several study on children [93,94]. However, insufficient physical activity and excessive sedentary behaviors are associated with functional constipation in Asian adolescent [95]. Physical activity could reduce symptoms of functional constipation in the longer time. A high level of physical activity, established with an actigraph accelerometer in children with an average age of 25 months, allows to reduce the incidence of functional constipation in the fourth year of life. This result was obtained in children who practiced physical activity for more than 60 min/day [96]. Adult patients with IBS are suggested to engage in slow, low-intensity activities (cycling, swimming, yoga and aerobics) for 20 to 60 min three to five times a week. These kinds of physical activities have beneficial effects on gastrointestinal symptoms, particularly in reducing the occurrence of constipation, and the well-being of individuals with IBS [97]. Additionally, active individuals tend to have better eating habits, such as drinking more water and maintaining regular mealtimes [98]. In conclusion, physical activity combined with an adequate diet are two important therapeutic aspects of the management of overweight and gastrointestinal functional disorders. In addition, physical activity allows additional benefits, reducing symptoms of anxiety and depression [98,99].

Functional abdominal pain disorders (FAPDs), known as disorders of gut–brain interaction, are also related to emotional difficulties. Children and adolescents with FAPDs have higher levels of anxiety and depression, as well as lower quality of life, than healthy children [100]. Home and school related stressors are associated with the development of functional constipation in children [101]. Children and adolescents could have severe limitations in their social life and daily activities resulting from FAPDs due to the symptoms, and this can harm functional gastrointestinal disorders. In the management of patients with FBS, clinicians should instruct the patient to intervene psychologically, with active family participation and ongoing medical management, when they do not respond to conventional treatment [102]. Family interactions can influence the development and maintenance of FGIDs through their effects on the physical and psychosocial functioning of an individual [103].

Several studies show that cognitive behavioral therapy could play a crucial role in reducing intensity for children with pain, especially in abdominal migraine and abdominal functional pain [104,105]. A randomized controlled trial reported that children who received a combination of standard medical care and CBT, along with their parents, experienced significantly less abdominal pain than those who received medical care alone [106]. Additionally, a recent meta-analysis indicated that CBT effectively ameliorates functional abdominal pain, reduces pain intensity, and enhances physical quality of life in affected children [107].

These findings underscore the importance of integrating family-focused CBT approaches in managing pediatric FGIDs to achieve optimal therapeutic outcomes.

**Figure 2:**
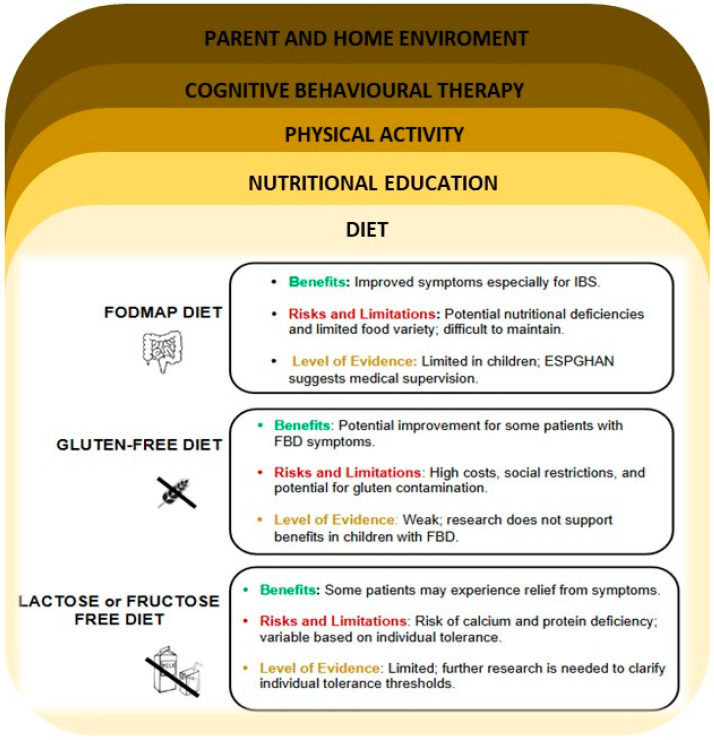
Dietary strategies and intervention areas useful in the management of children with FBDs.

Figure 2 summarizes the evidence regarding dietary strategies and represents the various intervention areas that should be considered in the management of children with FBDs (Figure 2).

Given the established link between obesity and FGIDs [15,16,17], it is plausible that weight management, including weight loss, could alleviate FGID symptoms in affected children. Implementing healthy lifestyle changes, such as balanced nutrition and regular physical activity, may not only reduce BMI but also potentially improve gastrointestinal function.

## 5. Gut Implication

The human gut microbiota is a vast ecosystem of microorganisms that play a central role in various physiological functions, including metabolism, immune modulation, and gut health [108].

Increasing evidence points to a complex and bidirectional relationship between the gut microbiota, FBDs, and obesity, particularly in children. Indeed, changes in gut microbiota content are associated with the onset and persistence of FBDs as well as have been described as an important determinant in the pathophysiology of obesity [109]. When FBDs and obesity coexist, the altered microbiota tends to amplify dysfunctions present in each condition, worsening both gastrointestinal and metabolic symptoms [109,110]. Obesity-related systemic inflammation, coupled with gut dysbiosis, may exacerbate gut permeability (‘leaky gut’) and visceral hypersensitivity, both hallmarks of FBDs. These mechanisms underline the need for interventions targeting the microbiome to simultaneously address obesity and FBD symptoms.

### 5.1. The Impact of Gut Microbiota

#### 5.1.1. Dysbiosis in Functional Bowel Disorders and Obesity

Gut microbiota composition in both FBDs and obesity is typically disrupted, showing reduced diversity and an overabundance of certain proinflammatory bacteria [111].

The balance between bacterial populations, rather than their absolute quantities, often plays a more critical role in shaping the functional impact of the gut microbiota. For instance, shifts in the Firmicutes-to-Bacteroidetes ratio have been highlighted as key determinants in obesity and related conditions [112,113]. Although the composition of dysbiotic microbiota is still controversial, some studies reported that children with obesity have higher levels of *Firmicutes* and lower levels of beneficial bacteria such as *Bacteroidetes* and *Bifidobacteria*, resulting in an enhanced capacity to derive energy from food due to a more active breakdown of carbohydrate complexes [112,113,114]. The composition of gut microbiota in FBDs is controversial, but patients seem to have a similar dysbiosis status [115]. As the gut microbiota is responsible for several mechanisms (e.g., the fermentation of undigested proteins and carbohydrates, the utilization of hydrogen, the transformation of bile acids and gas), dysbiosis can lead to disturbances of the intestine’s immune function, altered motility and gut permeability, commonly referred to as “leaky gut,” allowing bacterial components to enter the bloodstream [116]. The immune system activation associated with this process further exacerbates gut-related symptoms and can contribute to a chronic inflammatory state, often observed in individuals with FBDs [116]. These changes lead to worsened gastrointestinal symptoms, such as bloating, abdominal pain, and constipation [27].

#### 5.1.2. Microbiota-Mediated Mechanisms and Therapeutic Implications

A study by Saulnier et al. [117] showed that the content of microbiota correlated with the severity and rate of abdominal pain in children with IBS. These symptoms may be intensified due to changes in microbiota-mediated production of short-chain fatty acids (SCFAs) and gasses like methane, which have been shown to affect gut motility and sensitivity [118]. Even the relationship between gut microbiota and FC is frequently explored through its impact on gut transit time. This could be due to microbial influence on gene expression that affects the gut’s motor responses, pH-dependent motility stimulation by fermentation, osmotic effects caused by microbial metabolites, and intestinal stretching due to increased intraluminal gas production, which triggers reflexive contractions of the smooth muscle [119,120]. The differences in the gut microbiota between children with FC and the control group were shown in different studies [119,120]. One study evidenced how children with obesity and FC had significantly lower levels of *Bacteroidetes*, in particular *Prevotella*, and higher levels of some groups of *Firmicutes*, such as Lactobacillus, compared with healthy children [120]. Other studies also highlighted increased levels of *Bifidobacteria* [119,121].

The coexistence of altered gut microbiota, obesity, and FBDs may have an impact on the efficacy of dietary and pharmacological interventions. For example, dietary modifications commonly recommended for FBDs, such as the low-FODMAP diet, may affect the microbiota composition by temporarily reducing bacterial fermentation [122]. The efficacy of specific probiotics, such as *Lactobacillus reuteri* and *Bifidobacterium* species, varies due to strain-specific mechanisms affecting motility and gut barrier integrity. For instance, while *Lactobacillus reuteri* DSM 17,938 showed benefits in some studies, others found limited effects on stool frequency, suggesting that patient-specific factors like baseline microbiota composition may influence outcomes [123]. Personalized interventions related to gut microbiota composition may be essential for improving clinical outcomes in children affected by both conditions.

### 5.2. The Role of Biotics and Integrative Support

#### 5.2.1. Biotics

Given the role of diet in modulating gut microbiota and influencing symptoms of FBDs, additional strategies like probiotics and prebiotics hold promise in optimizing gut health and symptom management. The WHO defines probiotics as “live microorganisms which when administered in adequate amounts confer a health benefit on the host” [124]. Noteworthy, to be defined as probiotics, microorganisms must demonstrate evidence-based health benefits [125]. Probiotics have demonstrated effectiveness in preventing and/or treating many gastrointestinal disorders, such as infectious and antibiotic-associated diarrhea, ulcerative colitis, infantile colic, IBS, and FAP [126]. They interact with the host through mechanisms such as enhancing gut barrier function, modulating immune responses, and influencing microbial composition. The activity of probiotics is strain and species-specific and changes according to the physiology and eating habits of different subjects [127]. Specific probiotics can directly act on gut motility and barrier functions, host digestion, immune responses, nociception, metabolism, behavior, and microbiota composition [126]. Concerning functional constipation, a randomized control trial (RCT) involving 45 children over a 4-week protocol, evaluated the effects of *Lactobacillus casei rhamnosus* (Lcr35) on abdominal pain and intestinal transit. The authors compared the efficacy of Lcr35 with magnesium oxide (MgO) and placebo [128]. While improvements in abdominal pain and intestinal transit were observed, the results were not statistically significant. Furthermore, the efficacy of *Lactobacillus reuteri* DSM 17,938 has been assessed in several studies [128,129,130]. Kubota and colleagues also found a reduction in the abundance of the genus *Dialister* in the group of children administrated with the MgO, an osmotic laxative frequently used in constipation treatment, suggesting that further studies are needed to explore better the interplay between the treatment and the gut microbiota [129]. Besides, the results have been inconsistent so far.

Prebiotics are substrates selectively used by the host microbiota. These molecules exert a positive impact on intestinal barrier function by modulating tight junctions (TJs) through their interaction with the gut microbiota, providing health benefits [131]. Molecules that have been tested for treating FBDs include inulin, galacto-oligosaccharides (GOS), fructo-oligosaccharides (FOS), glucomannan, psyllium, and different fiber mixtures [131].

Lately, interest in the use of inulin in the treatment of FC has been rising. Closa-Monasterolo et al. showed that administering 2g/day of inulin for six weeks significantly improved stool consistency [132]. Lohner et al., in a cohort of children aged 3 to 7 years receiving 6 g/day of inulin for 24 weeks, demonstrated that the relative abundance of *Bifidobacterium* and *Lactobacillus* in stool samples of children receiving fructans, was respectively 19.9% and 7.8% higher compared to the control group at week 24 (*p* = 0.014 for *Lactobacillus* and *p* < 0.001 for *Bifidobacterium*). Also, children in the treated group experienced significantly softer stools, starting from week 12 [133].

Recent studies indicate that certain prebiotics may have a role in regulating mucus production, composition, and degradation. FOS has been studied as prebiotics in both animals and humans. These molecules may improve glucose, lipid, and energy metabolism in the host, by modifying the composition of the gut microbiota [134,135]. For instance, the administration of FOS has been shown to ameliorate metabolic disorders resulting from a high-fat diet by promoting the secretion of glucagon-like peptides 1 and 2 (GLP-1 and GLP-2) [136,137].

Research explored the effect of fiber mixtures at different dosages in pediatric populations. In particular, Kokke et al. studied the impact of a fiber blend (including soy fiber, inulin, GOS, and resistant starch) compared to lactulose in 97 children with FC [138]. However, no significant differences were observed in defecation frequency or abdominal pain. When compared to polyethylene glycol (PEG), a fiber mixture comprising acacia fiber, psyllium fiber, and fructose, yielded comparable results after 8 weeks in terms of bowel movement frequency, stool consistency, and fecal incontinence [138]. One systematic review and meta-analysis of three RCTs investigated the efficacy of glucomannan in the improvement of the symptoms in children with FC [139]. Staiano and colleagues reported a statistically significant improvement in stool consistency after a treatment of 12 weeks with 100 mg/kg of glucomannan in a cohort of 20 children (*p* < 0.01) [140].

Dietary fiber supplementation is a common approach for managing IBS, but its effectiveness can vary based on the type of fiber. Soluble fibers, found in some fruits and legumes, absorb water, slow down intestinal transit, and reduce cholesterol absorption [85]. In contrast, insoluble fibers, found in fruit peels and bran, increase fecal volume and accelerate intestinal transit, making them more suitable for IBS patients who experience constipation [141]. Baştürk et al. conducted an investigation using a synbiotic formulation that included the probiotic strains *Lactobacillus casei*, *Lactobacillus rhamnosus*, *Lactobacillus plantarum*, and *Bifidobacterium lactis*, with diverse prebiotics, including fiber, polydextrose, FOS, and GOS. After 4 weeks of treatment, the overall benefits observed from the synbiotic intervention were significantly greater than those in the placebo group [142]. Although especially probiotic supplements have shown significant improvements in abdominal symptoms compared to placebo in patients with IBS, there is still a lack of long-term evidence for their use, particularly in children [141].

#### 5.2.2. Curcumin and Other Phytochemicals

Polyphenols, such as catechins, anthocyanins, curcumin, have shown promising effects on lipid metabolism, energy regulation, and weight management [143]. Animal and cell studies propose several mechanisms behind these benefits, including the inhibition of adipocyte differentiation, enhanced fatty acid oxidation, reduced fatty acid synthesis, stimulation of thermogenesis, facilitation of energy metabolism, and inhibition of digestive enzymes [144]. Anthocyanins also exhibit antibacterial properties, inhibiting the growth of pathogens like *Bacillus cereus* and *Helicobacter pylori*, suggesting their role in modulating gut microbiota that may contribute to obesity and inflammatory disease management [145].

Curcumin, recognized for its antioxidant properties, is one of the most studied polyphenol [144]. In a meta-analysis of RCTs, has been shown that curcumin reduce oxidative stress in a dose- and duration-dependent manner by scavenging free radicals and inhibiting the production of malondialdehyde [146]. Specifically, curcumin significantly reduced malondialdehyde [SMD −0.46 (CI 95%: −0.68 to −0.25)] and increased superoxide dismutase [0.82 (0.27 to 1.38)], glutathione peroxidase [8.90 (6.62 to 11.19)], and catalase [10.26 (0.92 to 19.61)], comparing with control group. Also, the co-administration of piperine and curcumin may have a potential to enhance curcumin’s effectiveness in supporting the antioxidant molecules role [146]. Once curcumin reaches the intestines, it is metabolized by the host’s microbiota. This process produces secondary metabolites that may promote the growth of beneficial bacterial strains in the host gut [147]. Additionally, curcumin has been studied as potential support in alleviating IBS symptoms other than metabolic disease and obesity [148], enhancing pain relief and quality of life, although the results are not consistent, particularly in children [149,150].

However, despite its therapeutic potential across several diseases, curcumin’s clinical utility is limited by its poor gastrointestinal absorption and low bioavailability [151]. These limitations are mainly due to its insolubility in water, rapid metabolism, and excretion [152]. However, recent evidence suggest a need to redefine bioavailability because of the gut microbiota’s role as a dynamic organ that interacts with and processes curcumin and other molecules [153,154]. Hassaninasab et al. demonstrated that *Escherichia coli* is the microorganism with the highest ability to metabolize native curcumin, leading to obtain tetrahydrocurcumin, the reduced analog of curcumin predominantly present in the gastrointestinal tract [147]. Furthermore, *Bifidobacterium longum*, *Lactobacillus acidophilus*, *Bifidobacterium pseudocatenulatum*, and *Lactobacillus casei*, have been shown to metabolize curcumin, reducing over 50% of the original compound, emphasizing their role in converting curcumin into bioactive metabolites that could enhance its physiological effects [155,156].

Despite curcumin’s good tolerability and its antioxidant and anti-inflammatory properties [157], evidence supporting its efficacy is still limited and its effect mainly comes from studies conducted on adult populations [158].

In Table 2, the summary of the papers referred to dietary interventions and integrative support in children with FBDs are resumed.

## 6. Limitations

We recognize several limitations in this review. First, this is a narrative review, which offers a non-systematic overview and analysis of the existing literature on the topic [15,27]. The absence of standardized guidelines for narrative reviews can lead to selection biases and often results in qualitative rather than quantitative analyses. For example, our review focused solely on articles available on PubMed, which may have excluded relevant studies indexed in other databases or published in non-English languages. This may have limited the diversity and comprehensiveness of the included studies.

Another limitation is the scarcity of long-term follow-up data in the available studies. Most of the reviewed evidence lacks robust longitudinal assessments leaving significant gaps in understanding how therapeutic approaches influence the progression and management of FGIDs over time. This limitation highlights the need for future studies with standardized follow-up protocols to evaluate the sustainability of interventions and their long-term impacts on pediatric populations.

Additionally, data on integrative support in pediatrics remain heterogeneous, with considerable variability in study designs, populations, and intervention protocols. The inconsistency of findings regarding the efficacy of probiotics, prebiotics, and other dietary interventions underscores the need for large-scale randomized controlled trials to establish evidence-based recommendations.

Furthermore, the complex interplay between gut microbiota, dietary factors, and psychosocial elements presents another challenge. Many studies do not adequately account for potential confounders, such as genetic predispositions, which may influence the outcomes and limit the generalizability of findings. Socioeconomic status, physical activity levels, and dietary patterns can act as confounders in studies linking obesity and FBDs. For example, sedentary behavior not only contributes to obesity but also influences gastrointestinal motility, underscoring the importance of multifaceted approaches in both research and clinical management.

To deepen our understanding of the relationship between of obesity and FGIDs, it will be crucial to integrate prevention and treatment strategies into broader frameworks that account for these multifactorial influences. Future research should prioritize interdisciplinary collaboration and aim to address these limitations by utilizing more comprehensive study designs, expanding inclusion criteria, and embracing innovative methodologies, such as microbiome analyses and machine learning models for data integration.

## 7. Conclusions

A potential association between obesity and FGIDs has been identified with both conditions sharing common characteristics, including high prevalence in children, diet- and lifestyle-related risk factors, gut microbiota imbalances, and psychological challenges. Managing this dual condition in pediatric patients presents unique challenges, particularly due to poor adherence to conventional treatments. This narrative review emphasizes the need for holistic and multidisciplinary approaches to symptom management. Nutrition education, physical activity, and medical care must be complemented by additional strategies such as psychological interventions and personalized dietary modifications, including low-FODMAP and fiber-enriched regimens. These strategies not only address immediate symptoms but also provide a foundation for long-term health benefits. Considering the interplay between gut microbiota alterations, obesity, and FGIDs, the modulation of gut microbiota through probiotics, prebiotics, and integrative support shows significant promise. Although current evidence highlights their potential in prevention and treatment, the variability in findings calls for robust, well-designed longitudinal studies to establish standardized protocols and maximize their efficacy. Addressing this issue holistically and combining these approaches will not only enhance the scientific understanding of the connection between obesity, gut microbiota, and FBDs but also improve the quality of clinical and preventive care for pediatric patients. Future research should aim to elucidate the bidirectional mechanisms linking these conditions, focusing on personalized interventions tailored to individual gut microbiota profiles. Standardization of diagnostic criteria and measures of intervention efficacy is crucial to facilitate the reproducibility and applicability of findings.

Ultimately, bridging the gaps in knowledge and practice through an integrated, evidence-based framework has the potential to improve patient outcomes and deepen our understanding of the intricate relationship between metabolic and gastrointestinal health in children.

## Figures and Tables

**Table 1:** Summary of the papers referred to state of the problem about Functional Gastrointestinal Disorders (FGIDs) in children and adolescents with obesity.

Author, Year	Study Design	Sample	FBDs	Age	Main Results
Teitelbaum J et al., 2009 [22]	Randomized-controlled trial	1012 (*n* 757 patients and *n* 255 controls)	FC, GE, IBS, encopresi and FAP, gastroesophageal reflux, irritable bowel syndrome, encopresis, and functional abdominal pain	2–20	Significantly higher prevalence of obesity in children with IBS
Galai T et al., 2020 [26]	Retrospective study	173 partecipants	FAP	2–18	Statistically significant higher prevalence of obesity or overweight compared to the control group
Tambucci R et al., 2019 [27]	Case-control study	103 partecipantswith FAP and 115 controls	FAP, FD, IBS, FC	4–18	A difference was noted between the two groups for FC, FD, and IBS but no statistically significant difference was observed for FAP
Phatak UP et al., 2014 [28]	Comparative study	450 participants	IBS	4–18	Overweight children have a higher prevalenc of FGIDs than normal-weight children
Pashankar DS et al., 2005 [29]	Retrospective analysis	719 partecipants	FC	4–18	No significant difference between children with obesity and normal weight children
Zia JK et al., 2022 [30]	Review	348 studies	FGIDs	No age limits	It’s necessary to implement studies on the pediatric population to determine the risk factors of FGIDs
Bonilla S et al., 2011 [32]	Prospective cohort study	188 partecipants	AP	9–18	Obesity predicts persistence of AP in children with FGIDs
Elitsur Y et al., 2009 [31]	Retrospective analysis	738 partecipants	GERD	2–18	No significant difference between childern with obesity and normal weight children
Patel N et al., 2010 [33]	Retrospective analysis	230 partecipants	GERD	2–20	No significant difference between children with obesity and normal weight children
Meng D et al., 2023 [34]	Review	165 studies	Relationship between the microbiome, obesity and functional disorders	No age limits	Exercise induces changes in the intestinal microbiome, regulating it and reducing the various disorders associated with it

Legend: Functional Constipation (FC); Gastroesophageal Reflux (GE); Irritation Bowel Syndrome (IBS); Functional Abdominal Pain (FAP); Gastroesophageal Reflux Disease (GERD); Abdominal Pain (AP), Functional Dyspepsia (FD).

**Table 2:** Summary of the papers referred to dietary interventions and integrative support in children with FBDs.

Author, Year	Study Design	Sample	FBDs	Age	Main Results
Dietary interventions					
Katsagoni CN et al. 2023 [60]	Systematic review	15 included articles. No weight information	FGIDs	Age: 3–18 years	Low-FODMAP diet, LRD, FRD, and GFD have no place in daily clinical practice for the management of children and adolescents with FGIDs. Nevertheless, some patients with IBS or RAP may experience some benefit from the use of a low-FODMAP diet or FRD/LRD. Limited data suggest that MD may be promising in the management of FGIDs, especially in IBS patients, but more data are required
El Ezaby SA et al. 2024 [61]	Randomized Controlled Trial	80 participants (*n* = 39 intervention group; *n* = 41 control group) Normal weight: 68% Overweight: 14% With obesity: 5%	IBS	Intervention group: 10.33 ± 2.80 years Control group: 10.98 ±2.59 years	Low FODMAP dietary intervention in children with IBS for six weeks decreased abdominal pain severity, improved gastrointestinal symptoms, and improved the health-related quality of life
Tenenbaum RB et al. 2023 [62]	Randomized Controlled Trial	30 participants (27% with obesity)	AP	Age: 7–12 years	Greater baseline quality of life was associated with better adherence to the low FODMAP dietary intervention (beta coefficient β = −0.2; *p* = 0.03); while abdominal pain complaints were not significantly associated with adherence.
Beleli CA et al. 2015 [73]	Non-randomized interventional double-blinded, placebo-controlled, crossover assignment study	20 participants No information on weight	FC	Age: 4–16 years	Galactooligosaccharide ingestion was related to increase of the bowel movement frequency (*p* < 0.0001), relief of defecation straining (*p* < 0.0001) and decrease in stool consistency (*p* = 0.0014), compared to placebo ingestion.
Dehghani SM et al. 2019 [74]	Randomized controlled double blinded trial	86 participants (*n* = 45 PEG syrup group; *n* = 41 BSM syrup group) no weight information	FC	Age: 4–12 years	BSM and PEG syrups had similar efficacy on FC.
Tajik *p* et al. 2018 [75]	Randomized Controlled Trial	60 participants (*n* = 30 intervention group; *n* = 30 control group). No weight information	FC	Age: 2–10 years	No significant difference was found between effects of red sugar and fig syrup in terms of the frequency of fecal excretion, and pain at the time of excretion (*p* = 0.264). Red syrup was more effective in reducing abdominal pain compared with fig syrup (*p* < 0.001), while fig syrup was more effective in inducing diarrhea (*p* = 0.019).
de Bruijn CM et al. 2022 [83]	Systematic review and meta-analysis	12 included articles, 819 participants 1 study included children with obesity	FAPDs	Age: 4–18 years	Very low-certainty evidence that the use of fibers leads to higher treatment success was found.
Alfaro-Cruz L et al. 2020 [87]	Narrative review	21 included articles No weight information	FAPDs	NA	Inconsistent results for the efficacy of low-FODMAP diet on FBDs in children
Inan M et al. 2007 [93]	Cross-sectional and descriptive study	1900 participants No weight information	FC	7–12 years	A negative correlation between physical activity and functional constipation
Chien LY et al. 2011 [94]	Observational study	14,626 participants Normal weight: 75.8% Overweight: 14.9% With obesity: 5.9%	FC	7–12 years	More time spent on sedentary activity was independently associated with increased risk of low defaecation frequency.
Huang R et al. 2014 [95]	Observational study	26,864 participants No weight information	FC	11–18 years	Constipation was associated with insufficient exercise and excessive sedentary behaviors
Driessen LM et al. 2013 [96]	Population-based prospective cohort study	347 participants No weight information	FC	Age: <4 years	Physical activity could reduce symptoms of functional constipation in the longer time. A high level of physical activity, established with an accelerometric actigraph in children with an average age of 25 months, allows to reduce the incidence of functional constipation in the fourth year of life. This result was obtained in children who practiced physical activity for more than 60 min/day
Chen JY et al. 2023 [105]	Systematic review and meta-analysis	10 included articles, 872 participants 1 study with children with obesity	FAPDs	Age: <18 years	CBT had significantly positive effects on reducing pain intensity, the severity of gastrointestinal symptoms, depression, and solicitousness and improved the quality of life decreasing the total social cost.
Integrative support					
Bu LN et al. 2007 [128]	Double-blind placebo-controlled, randomized study	45 participants: group A (*n* = 18) with MgO (50mg/kg/die); group B (*n* = 18) with Lcr35, 8 × 10 ^8^ c.f.u./die; group C (*n* = 9) with placebo No weight information	Chronic constipation	Age: <10 years	MgO and probiotics resulted in significant improvements over the placebo group in terms of increased stool frequency (*p* = 0.03), higher treatment success rate (*p* = 0.01), less use of glycerin enemas (*p* = 0.04), and softer stools (*p* = 0.01). There were no significant differences between MgO and probiotics. MgO showed a slightly earlier effect on constipation (week 2) than probiotics (weeks 2 to 3).
Kubota M et al. 2020 [129]	Double-blind, placebo-controlled, randomized, and parallel-group trial	60 participants; group A (*n* = 20) with L. reuteri DSM 17,938 and lactose hydrate as a placebo of MgO; group B (*n* = 19) with L. reuteri DSM 17,938 and MgO; and group C (*n* = 21) with a placebo of L. reuteri DSM 17,938 and MgO No weight information	FC	Age: 6 mo–6 years	Significant improvement in defecation frequency by the 4 week compared to baseline (group A: *p* < 0.05; group B: *p* < 0.05; group C: *p* < 0.05). The MgO group and the combination group registered a reduction in stool consistency, whereas the L. reuteri DSM 17,938 group did not (group A: *p* = 0.079; group B: *p* < 0.05; group C: *p* < 0.05).
Closa-Monasterolo R et al. 2017 [132]	Double-blind, randomized, placebo-controlled parallel group trial	17 participants; inulin-type fructans group (*n* = 8); placebo group (*n* = 9) No weight information	FC	Age: 2–5 years	No significant changes in the control group; the supplemented children showed an improvement in stool consistency (from 2.2 to 2.6 on the modified Bristol scale for children) (*p* = 0.040).
Lohner S et al. 2018 [133]	Randomized, parallel, double-blind, placebo-controlled trial	219 participants No weight information	FC	Age: 3–6 years	The relative abundance of Bifidobacterium (*p* < 0.001) and Lactobacillus (*p*= 0.014) were 19.9% and 7.8% higher, respectively, in stool samples of children receiving fructans compared to the control group at week 24. This was also associated with significantly softer stools, in the prebiotic group (from week 12).
Kokke FT et al. 2008 [138]	Randomized, double-blind, prospective controlled study	135 participants; fiber mixture group (*n* = 65); lactulose group (*n* = 70) No weight information	FC	Age: 1–13 years	No significant differences were found between the groups regarding defecation frequency (*p* = 0.481) and fecal incontinence (*p* = 0.084). Stool consistency was notably softer in the lactulose group (*p* = 0.01). Scores for abdominal pain and flatulence were comparable (*p* = 0.395 and *p* = 0.739).
Staiano et al. 2000 [140]	Randomized, double-blind trial	20 participants; glucomannan group (*n* = 10); placebo group (*n* = 10) No weight information	Chronic constipation	Age: 5.7 ± 4.2 (mean ± SD) years	Glucomannan significantly increased stool frequency (*p* < 0.01). It also notably reduced the use of laxatives or suppositories (*p* < 0.01) and improved stool consistency, with a significant reduction in painful defecation episodes per week (*p* < 0.01).
Baştürk A et al. 2017 [142]	Randomized, controlled trial	146 participants; symbiotic group (*n* = 72); placebo group (*n* = 74) No weight information	FC	Age: 4–18 years	Significant improvement of frequency of defecations, abdominal pain, painful defecation, and pediatric Bristol scale scores (*p* ≤ 0.001) in the synbiotic group. Complete treatment benefits were achieved by 66.7% patients in the synbiotic group and 28.3% patients in the placebo group, with a significant difference between groups (*p* ≤ 0.001).

Legend: Black Strap Molasses (BSM); Cognitive Behavioral Therapy (CBT); Irritable Bowel Syndrome (IBS); Functional Abdominal Pain Disorders (FAPDs); Functional Bowel Disorders (FBDs); Functional Constipation (FC); Functional Gastrointestinal Disorders (FGIDs); Fermentable Oligosaccharides Disaccharides Monosaccharides And Polyols (FODMAP); Fructose- Or Lactose-Restricted Diet (FRD or LRD); Gluten-Free Diet (GFD); low-FODMAP diet (LFD); Mediterranean diet (MD); Not Available (NA); Lactulose And Polyethylene Glycol (PEG); Recurrent Abdominal Pain (RAP); Magnesium Oxide (MgO).

## Data Availability

Not applicable.
